# Retroperitoneal Ganglioneuroma in a Patient Presenting With Vague Abdominal Pain

**DOI:** 10.7759/cureus.9133

**Published:** 2020-07-11

**Authors:** Malik Hatim Hussain, Zafar Iqbal, Muhammad Shoaib Mithani, Muhammad Noman Khan

**Affiliations:** 1 Orthopaedics and Trauma, East Lancashire NHS Hospitals, Blackburn, GBR; 2 Emergency Medicine, California Institute of Behavioural Neurosciences and Psychology, Fairfield, USA; 3 Emergency Department, The Kidney Center, Karachi, PAK; 4 Urology, The Kidney Center, Karachi, PAK; 5 Emergency Medicine, Usman Memorial Hospital, Karachi, PAK

**Keywords:** neural crest cell tumor, ganglioneuroma, retroperitoneal tumor

## Abstract

Ganglioneuroblastoma, neuroblastoma, and ganglioneuroma (GN) are the tumors that arise from the neural crest cells. Of these, GN has the most benign origin without metastatic potential. The most common sites of their origin are the posterior mediastinum and retroperitoneum. Although the imaging studies, including CT, are available to detect these tumors, the definitive diagnosis can only be made by histological examination. We present a case of a 40-year-old woman with a retroperitoneal GN causing longstanding, gradually increasing, uncontrolled abdominal pain due to its pressure effect on the pancreas, duodenum, and right kidney with the displacement of the inferior vena cava. An exploratory laparotomy was performed, and the mass was removed. Histopathology confirmed the benign nature of the mass (a GN). These tumors are rarely malignant and mostly asymptomatic. However, in our case, abdominal pain was affecting the patient’s life. After a discussion with the patient, an elective surgical procedure was performed, and the patient was symptom-free postoperatively and able to resume her regular routine.

## Introduction

Ganglioneuromas (GN) originate from the neural crest tissue and are composed of gangliocytes and mature stroma. They are very rare and benign in their course [1]. The posterior mediastinum (in 39% to 43% of cases) and retroperitoneum (in 32% to 52% of cases) are the most common sites of presentation [2]. These tumors are usually an incidental finding, as most patients remain asymptomatic [3]. The tumor may cause pressure symptoms due to its large size [4]. Some GNs have been reported to be hormonally active, secreting catecholamines, vasoactive intestinal polypeptides, or androgenic hormones, which manifest with symptoms like hypertension, diarrhea, and virilization [5,6]. We present the case of a 40-year-old woman with GN.

## Case presentation

**Figure 1:**
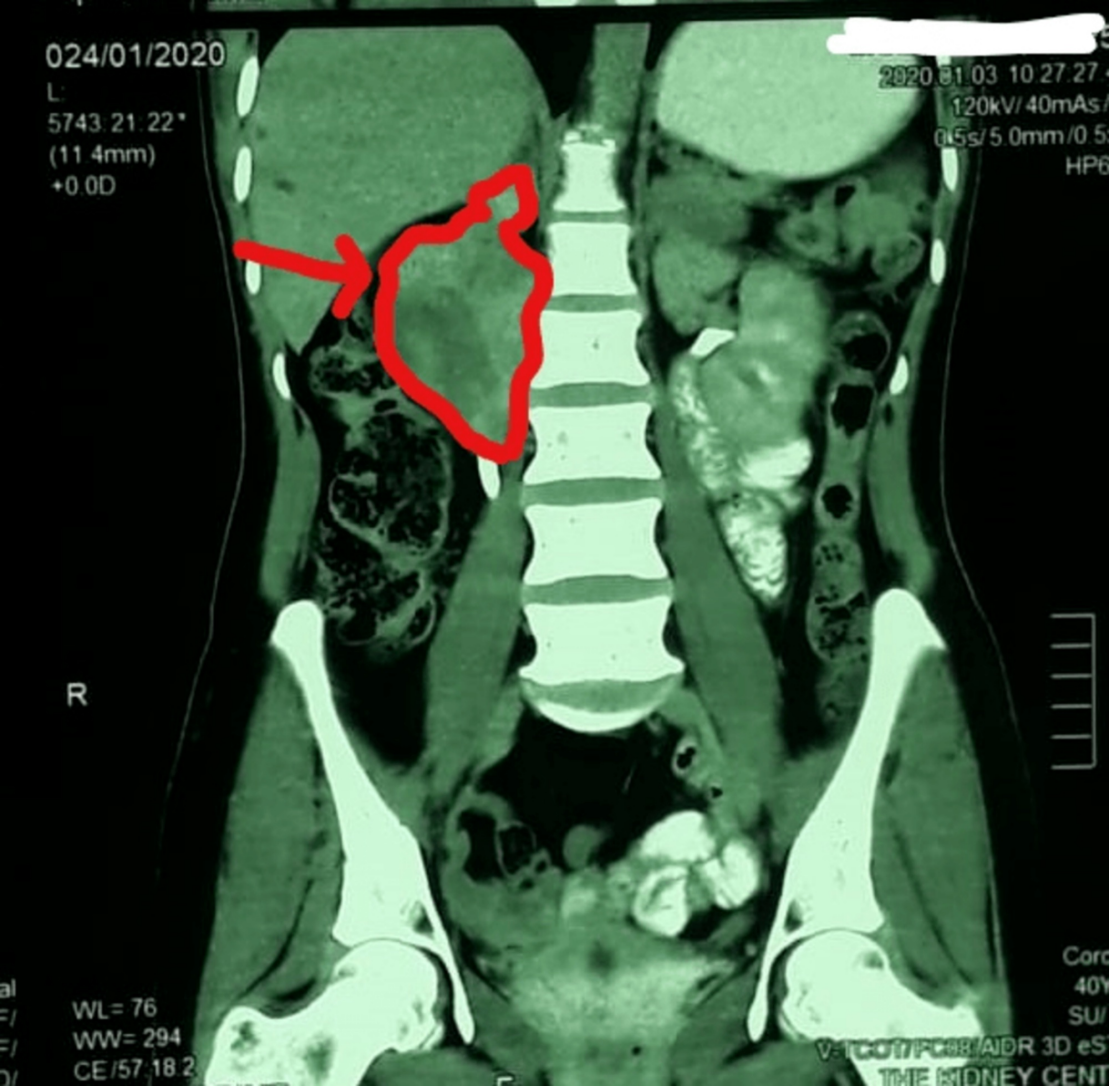
CT scan (coronal view) of the abdomen showing a right-sided mass.

**Figure 2:**
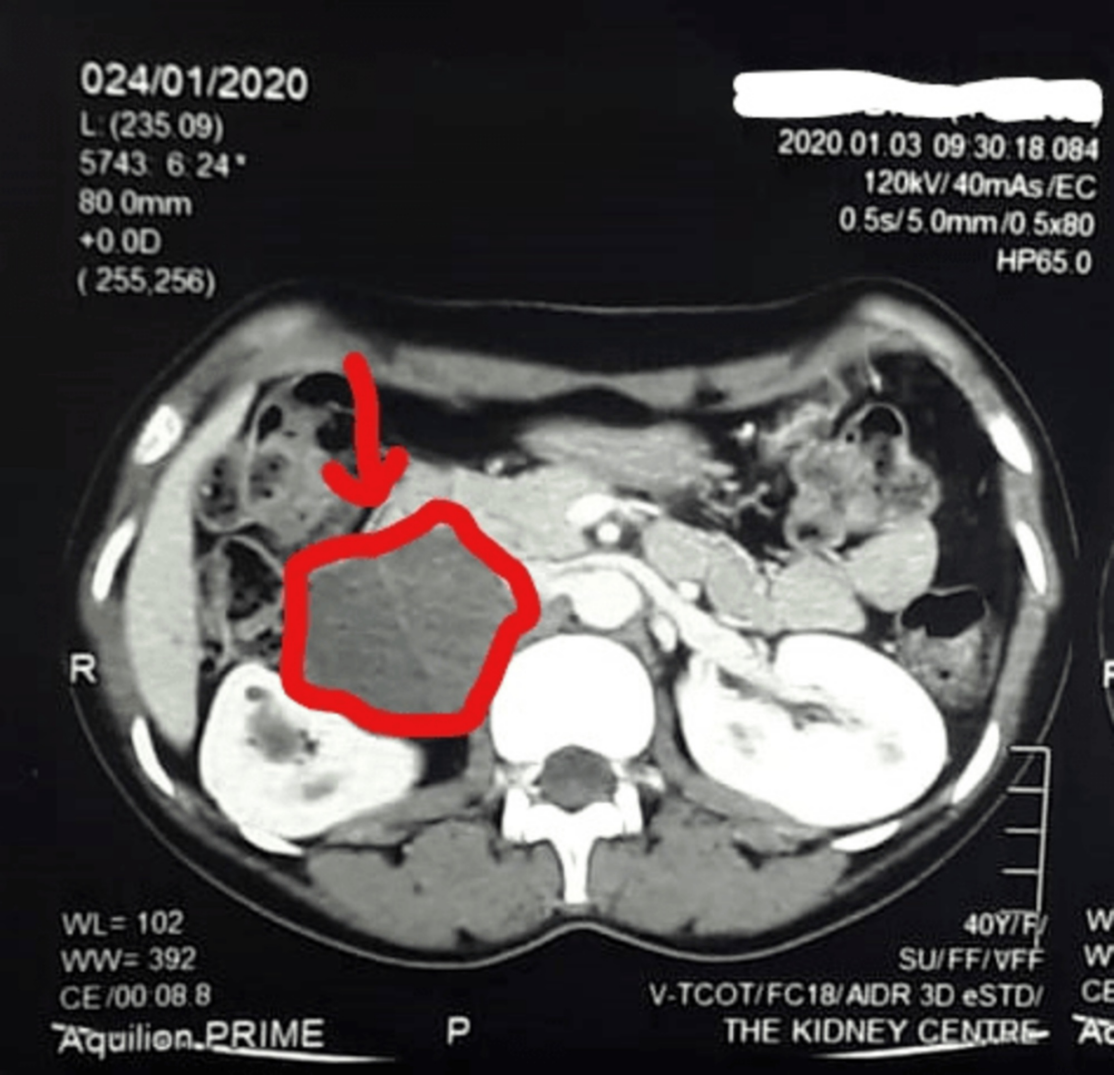
CT scan (axial view) of the abdomen showing a right-sided mass.

A 40-year-old woman presented to the hospital with vague, gradually increasing abdominal pain for the past two to three months. She denied radiation of pain and nausea/vomiting. Her last menstrual period was a week prior to presentation, and her past medical history and family history were noncontributory. She had no evidence of an acute abdomen. On physical examination, the abdomen was soft and mildly tender, and the rest of the physical findings were unremarkable. During her stay in the hospital, routine investigations were performed, and the results of which were within reference ranges. Urine dipstick and culture were negative. A pregnancy test was negative. A contrast-enhanced CT scan of the whole abdomen showed a retroperitoneal, well-circumscribed soft tissue density lesion containing subtle fluid attenuation areas and internal septation on the right side (Figures 1, 2).

The lesion measured 6.8 x 5.4 x 5.0 cm in craniocaudal, transverse, and anteroposterior dimensions, respectively. In addition, it was abutting the right kidney, compressing it. It was also indenting the duodenum and pancreas and slightly displacing the inferior vena cava without frank invasion. It was decided to observe the patient.

On her subsequent visit approximately 3.5 months later, she was still experiencing pain; however, its intensity was increasing each day. Her physical examination showed moderate tenderness, and the rest of the findings and the laboratory investigations were insignificant. However, the repeated CT scan depicted a well-defined mixed density lesion in the retroperitoneum on the right side along with the renal pelvis closely abutting with the duodenum and inferior vena cava anteriorly without invasion measuring 8.5 x 5.3 x 5.0 cm. These findings were suggestive of a benign etiology, and the possible differential included gastrointestinal stromal cell tumor, neurofibroma, or nodal lesion.

The patient’s symptoms had progressed and were influencing her daily routine. Therefore, an exploratory laparotomy was planned, the lesion was excised, and the specimen was sent for histopathology. The report depicted features consistent with GN. Since then, the patient has been monitored via regular follow-up, and her symptoms have been alleviated.

## Discussion

GN contains mature sympathetic ganglion cells, Schwann cells, stroma, and nerve fibers along with fibrous tissue. The origin of GN tumor is usually from the great sympathetic chain, which extends from the base of the skull, neck, in the chest posterior mediastinum to the retroperitoneum along with the adrenal gland [6,7]. Such tumors are very rare and mostly found during the third and fourth decades of life [7]. These tumors can be found in the neck, heart, bones, adrenal medulla, intestine, and spermatic cord [7]. GN is best managed surgically through laparoscopic excision. A Japanese study indicated a good prognosis postoperatively [6]. GN is mostly found in women, with a female:male ratio of 3:2 [8]. The preoperative diagnosis of GN is often difficult [6]. Most of the time, GN remains asymptomatic; however, in this case, the patient had vague abdominal pain secondary to mass effect on the surrounding structures.

## Conclusions

GN is a benign tumor and generally asymptomatic, but occasionally can produce hormones and manifest with symptoms. Careful evaluation by imaging studies is vital for the diagnosis. A definitive diagnosis can be made by histopathological evaluation of the excised lesion. Overall, the prognosis is good following the surgical removal of the tumor.
